# Supplementary material for: Real-time non-contact cellular imaging and angiography of human cornea and limbus with common-path full-field/SD OCT
Source: Nat Commun. 2020 Apr 20;11:1868. doi: 10.1038/s41467-020-15792-x (PMC7171111; doi:10.1038/s41467-020-15792-x)
Supplement: Supplementary file 2 — Description of Additional Supplementary Files [file 41467_2020_15792_MOESM2_ESM.pdf]

Description of Additional Supplementary Files

File Name: Supplementary Movie 1
Description: 3D scheme of setup for eye tracking and defocusing correction

File Name: Supplementary Movie 2
Description: 3D scheme of setup for eye tracking and defocusing correction (close view, optics only)

File Name: Supplementary Movie 3
Description: Tracking of OCT peaks, estimating corneal position and validating defocusing correction

File Name: Supplementary Movie 4
Description: Comparison of imaging steadily moving ex vivo cornea with and without defocusing correction

File Name: Supplementary Movie 5
Description: Imaging ex vivo cornea moved with natural frequencies of the eye

File Name: Supplementary Movie 6
Description: Real-time imaging of central human cornea in vivo (example 1)

File Name: Supplementary Movie 7
Description: Real-time imaging of central human cornea in vivo (example 2)

File Name: Supplementary Movie 8
Description: Real-time imaging of peripheral human cornea in vivo

File Name: Supplementary Movie 9
Description: Real-time imaging of limbal human cornea in vivo

File Name: Supplementary Movie 10
Description: Blink and tear flow

File Name: Supplementary Movie 11
Description: Movement of tear film caused by half-blink

File Name: Supplementary Movie 12
Description: Blood flow underneath Palisades of Vogt and tracking of blood cells
