# Supplementary material for: Real-time non-contact cellular imaging and angiography of human cornea and limbus with common-path full-field/SD OCT
Source: Nat Commun. 2020 Apr 20;11:1868. doi: 10.1038/s41467-020-15792-x (PMC7171111; doi:10.1038/s41467-020-15792-x)
Supplement: Supplementary file 15 — Reporting Summary [file 41467_2020_15792_MOESM15_ESM.pdf]

nature research

Corresponding author(s): A. Claude Boccara
Last updated by author(s): 2019/05/19

Reporting Summary

Nature Research wishes to improve the reproducibility of the work that we publish. This form provides structure for consistency and transparency in reporting. For further information on Nature Research policies, see Authors & Referees and the Editorial Policy Checklist.

Statistics

For all statistical analyses, confirm that the following items are present in the figure legend, table legend, main text, or Methods section.

n/a	Confirmed	
☐	☒	The exact sample size (*n*) for each experimental group/condition, given as a discrete number and unit of measurement
☐	☒	A statement on whether measurements were taken from distinct samples or whether the same sample was measured repeatedly
☒	☐	The statistical test(s) used AND whether they are one- or two-sided<br>*Only common tests should be described solely by name; describe more complex techniques in the Methods section.*
☒	☐	A description of all covariates tested
☒	☐	A description of any assumptions or corrections, such as tests of normality and adjustment for multiple comparisons
☐	☒	A full description of the statistical parameters including central tendency (e.g. means) or other basic estimates (e.g. regression coefficient) AND variation (e.g. standard deviation) or associated estimates of uncertainty (e.g. confidence intervals)
☒	☐	For null hypothesis testing, the test statistic (e.g. *F*, *t*, *r*) with confidence intervals, effect sizes, degrees of freedom and *P* value noted<br>*Give P values as exact values whenever suitable.*
☒	☐	For Bayesian analysis, information on the choice of priors and Markov chain Monte Carlo settings
☒	☐	For hierarchical and complex designs, identification of the appropriate level for tests and full reporting of outcomes
☒	☐	Estimates of effect sizes (e.g. Cohen's *d*, Pearson's *r*), indicating how they were calculated

Our web collection on statistics for biologists contains articles on many of the points above.

Software and code

Policy information about availability of computer code

Data collection	Custom written code in Labview 2014 and Thorlabs SpectralRadar SDK. Details on the use of the software are specified in the Methods section.
Data analysis	Matlab R2017a for plots and angiography maps; ImageJ 1.51p for image display, for measurements of cell and nerve densities. Details on the use of the software are specified in the Methods section.

For manuscripts utilizing custom algorithms or software that are central to the research but not yet described in published literature, software must be made available to editors/reviewers. We strongly encourage code deposition in a community repository (e.g. GitHub). See the Nature Research guidelines for submitting code & software for further information.

Data

Policy information about availability of data

All manuscripts must include a data availability statement. This statement should provide the following information, where applicable:

- Accession codes, unique identifiers, or web links for publicly available datasets
- A list of figures that have associated raw data
- A description of any restrictions on data availability

The data that support the findings of this study are available from the authors on reasonable request.

Field-specific reporting

Please select the one below that is the best fit for your research. If you are not sure, read the appropriate sections before making your selection.

☒ Life sciences ☐ Behavioural & social sciences ☐ Ecological, evolutionary & environmental sciences

Life sciences study design

All studies must disclose on these points even when the disclosure is negative.

Sample size	Due to the exploratory nature of this study, the sample size was not chosen.
Data exclusions	No images within the acquired videos were excluded. Only frames before the video acquisition (during subject aligning) and after the video acquisition (during subject's resting with closed eyes) were removed.
Replication	Experiments were replicated in several human subjects.
Randomization	Due to exploratory nature of the study no groups were formed.
Blinding	Blinding was not applicable to this study.

Reporting for specific materials, systems and methods

We require information from authors about some types of materials, experimental systems and methods used in many studies. Here, indicate whether each material, system or method listed is relevant to your study. If you are not sure if a list item applies to your research, read the appropriate section before selecting a response.

Materials & experimental systems

n/a	Involved in the study	
☒	☐	Antibodies
☒	☐	Eukaryotic cell lines
☒	☐	Palaeontology
☒	☐	Animals and other organisms
☐	☒	Human research participants
☒	☐	Clinical data

Methods

n/a	Involved in the study	
☒	☐	ChIP-seq
☒	☐	Flow cytometry
☒	☐	MRI-based neuroimaging

Human research participants

Policy information about studies involving human research participants

Population characteristics	The human participants were:<br>male of age 26<br>female of age 36<br>male of age 24
Recruitment	The inclusion criteria for the healthy-volunteer study were as follows: (i) males and females older than 18 years of age; (ii) No previous history of anterior eye disorders
Ethics oversight	CPP (Comité de Protection de Personnes) Sud-Est III de Bron, ANSM (Agence Nationale de Sécurité du Médicament et des Produits de Santé) study number 2019-A00942-55

Note that full information on the approval of the study protocol must also be provided in the manuscript.
